# Mediterranean-DASH intervention for neurodegenerative delay diet adherence, physical activity, and cognitive function among community-dwelling older adults

**DOI:** 10.3389/fnut.2026.1947659

**Published:** 2026-10-28

**Authors:** Hui Li, Shuxian Yang, Yongjuan Wu, Xiaohua Wu, Kai Kang, Zhanfeng Liu, Lei Fan, Linqi Diao

**Affiliations:** 1 Institute for Prevention and Control of Chronic Non-communicable Diseases, Henan Provincial Center for Disease Control and Prevention, Zhengzhou, Henan, China; 2 Neihuang County Center for Disease Control and Prevention, Neihuang, Henan, China; 3 Mengzhou County Center for Disease Control and Prevention, Mengzhou, Henan, China

**Keywords:** cognitive function, interaction, MIND diet, older adults, physical activity

## Abstract

**Background:**

Evidence on the associations between the Mediterranean-Dietary Approaches to Stop Hypertension (DASH) Intervention for Neurodegenerative Delay (MIND) diet adherence, physical activity (PA), and cognitive function in Chinese older adults is limited. This study aimed to examine these associations and their potential interactions among community-dwelling older adults in Henan Province, China.

**Methods:**

Participants were recruited from 17 districts/counties in Henan Province, China, using the multistage stratified probability-proportional-to-size (PPS) sampling method. MIND diet adherence was assessed using a food frequency questionnaire (FFQ), and PA was assessed based on self-reported exercise frequency and duration. Cognitive function was measured using the Chinese version of the Mini-Mental State Examination (MMSE). Weighted generalized linear models (GLMs) were used to examine the associations between MIND diet adherence, PA, and their potential interaction and MMSE scores.

**Results:**

Higher MIND diet scores were significantly associated with higher MMSE scores (*β* = 0.259, 95% *CI*: 0.154–0.364, *p* < 0.001), whereas PA showed no significant independent association (*β* = 0.016, 95% *CI*: −0.017 to 0.049, *p* = 0.335). No significant interaction was observed between MIND diet adherence and PA regarding global cognitive function (*β* = −0.005, 95% *CI*: −0.013 to 0.002, *p* = 0.152). Domain-specific analyses showed significant associations between MIND diet scores and registration, attention and calculation, and recall (all *β* > 0, all *p* < 0.05). However, no significant interaction between MIND diet adherence and PA was observed for any domain (all *p* > 0.05).

**Conclusion:**

Higher adherence to the MIND diet was independently associated with better cognitive function among community-dwelling older adults in central China. These findings suggest that adherence to the MIND diet may be a practical strategy for promoting cognitive health in aging populations.

## Introduction

Population aging is accelerating globally, accompanied by a rising burden of chronic diseases among older adults. Dementia is currently the seventh leading cause of death worldwide and one of the major causes of disability and dependency among older adults. An estimated 57 million people worldwide are living with dementia (1). As the country with the world’s largest aging population, China faces enormous challenges in dementia prevention and management. Cognitive decline is a critical transitional stage between normal aging and dementia. A national cross-sectional survey reported that the prevalence rates of cognitive decline and dementia among Chinese adults aged ≥60 years were 15.5 and 6.0%, respectively (2). Cognitive decline imposes substantial emotional, social, and economic burdens on individuals, families, and the healthcare system (3). Currently, no effective curative treatment is available for dementia (4). Therefore, priority should be given to intervening on modifiable risk factors to curb disease progression. Among these factors, diet and PA have drawn extensive attention for their protective effects against cognitive decline and the onset of dementia.

The MIND diet is a dietary pattern that combines the Mediterranean diet and the DASH diet. Accumulating evidence has suggested that greater adherence to the MIND diet may help preserve cognitive function and reduce the risk of Alzheimer’s disease, the most common type of dementia (5, 6). The MIND diet emphasizes the consumption of brain-healthy foods such as leafy greens, berries, nuts, and whole grains while limiting the intake of red meat, butter, and sweets (7). These dietary components are believed to exert neuroprotective effects through mechanisms such as reducing oxidative stress, inflammation, and amyloid-beta deposition (8). Similarly, regular PA, including aerobic exercise, resistance training, and even low-intensity PA, can enhance neurogenesis, improve cerebral blood flow, and reduce systemic inflammation (9, 10). A meta-analysis has demonstrated that higher levels of PA are associated with a 20% reduction in the risk of developing dementia (11). Nevertheless, the optimal intensity, duration, and type of PA for cognitive protection remain to be clarified (12).

The combined effects of the MIND diet and PA are hypothesized to be synergistic, potentially yielding greater cognitive benefits than either factor alone. A healthy diet provides essential nutrients such as omega-3 fatty acids, antioxidants, and polyphenols to support brain function, which may amplify the cognitive benefits of PA (13, 14). Similarly, PA may improve the bioavailability of these nutrients and strengthen their neuroprotective properties (15). However, existing studies have yielded inconsistent findings due to methodological heterogeneity, leaving gaps in our understanding of how these two lifestyle factors jointly affect cognitive health (16). Moreover, the role of demographic and socioeconomic factors cannot be ignored. Age, education level, and socioeconomic status have all been shown to influence dietary patterns, PA levels, and cognitive outcomes (2). For instance, older adults with lower education levels or socioeconomic status may face barriers to accessing healthy foods or engaging in regular PA, potentially exacerbating their risk of dementia (17). Accordingly, this study aimed to examine the associations between MIND diet adherence, PA, and cognitive function and to explore their potential interactions among older adults aged ≥60 years in Henan Province, China.

## Methods

### Study design and participants

This cross-sectional study was conducted in Henan Province, China, in 2023. Detailed information on the study design has been described previously (18, 19). A multistage stratified probability-proportional-to-size (PPS) sampling method was used to collect high-quality information from a representative sample of older adults aged ≥60 years who had resided in the area for at least 6 months during the past year. Participants were excluded if they (i) were <60 years of age; (ii) resided in barracks, dormitories, or nursing homes; (iii) suffered from severe mental health disorders or had hearing impairment that prevented them from completing the questionnaire; or (iv) had severe paraplegia that prevented them from completing the biomedical measures of physical performance and body composition. A total of 17 districts/counties were sampled based on their urbanization rate (three districts and three counties with a higher urbanization rate and six districts and five counties with a lower urbanization rate). In each selected district/county, a total of three streets/towns were selected according to the number of older adults aged ≥60 years. Subsequently, two villages/communities were selected from each street/town using the same criterion. Finally, 90 participants aged ≥60 years were randomly selected from each village/community using simple random sampling (20). A total of 9,180 participants were randomly selected, of whom 9,147 were aged ≥60 years and completed the survey. Participants with missing MMSE scores, biologically implausible PA values (exceeding the 95th percentile, >42.0 h/week), or missing PA or dietary data were sequentially excluded. Finally, 8,681 participants were included in the analysis (Figure 1). The study was conducted in accordance with the Declaration of Helsinki, and the protocol was approved by the Ethics Committee of Henan Provincial Center for Disease Control and Prevention (2023-KY-006-02). All participants provided written informed consent.

**Figure 1:**
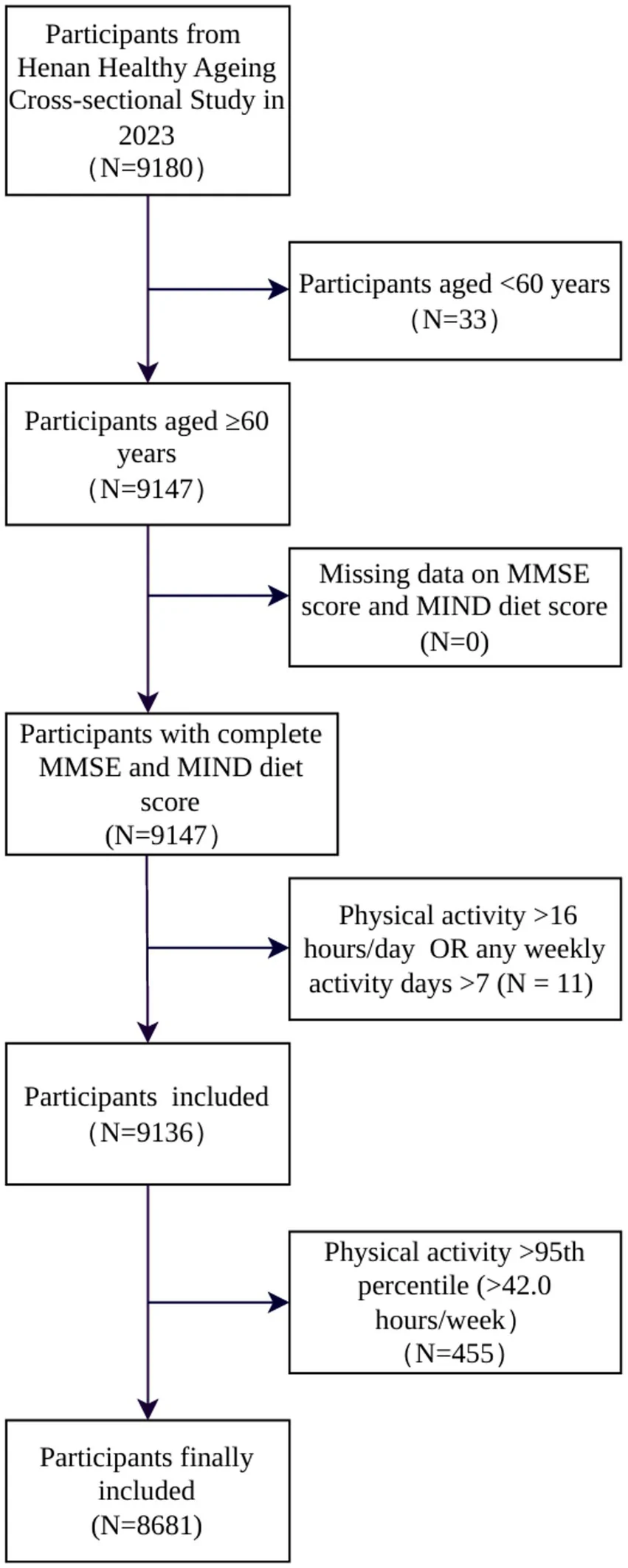
Flowchart of inclusion and exclusion.

### Cognitive function assessment

Cognitive function was assessed using the Chinese version of the MMSE, which includes 30 items covering five cognitive domains: orientation, registration, attention and calculation, recall, and language and visual construction skills. The total score ranges from 0 to 30, with higher scores indicating better cognitive function. MMSE assessments were administered using electronic questionnaires by trained staff.

### Assessment of the MIND diet

The MIND diet comprises 15 food groups: 10 groups with potential neuroprotective effects (leafy green vegetables, other vegetables, whole grains, beans, nuts, berries, poultry, seafood, olive oil, and wine) and five groups associated with adverse cognitive outcomes (red meat and products, fried foods, stick margarine and butter, cheese, and pastries and desserts). Dietary intake was assessed using a validated FFQ, and each food group was scored 0, 0.5, or 1 according to pre-defined cutoff values (Table 1) (7, 21). Whole grains, stick margarine and butter, and wine were scored based on daily intake. Beans, non-fried poultry, red meat products, and fried foods were scored by weekly intake frequency. Olive oil was scored according to whether it was the primary cooking oil used by the household. The remaining food groups were assessed based on weekly intake. The total MIND diet score was the sum of the scores across all 15 food groups.

**Table 1:** Component serving and scoring criteria for the MIND diet.

Diet component	0	0.5	1
Green leafy vegetables	<900 g/week	900–2,700 g/week	≥2,700 g/week
Other vegetables	1,250 g/week	1,250–1750 g/week	≥1750 g/week
Whole grains	<28 g/day	28–84 g/day	≥84 g/day
Beans	<1 meal/week	1–3 meals/week	>3 meals/week
Nuts	<7 g/week	7–140 g/week	≥140 g/week
Berries	<200 g/week	200–400 g/week	≥400 g/week
Poultry (not fried)	<1 meal/week	1–2 meal/week	≥2 meal/week
Seafood (not fried)	0	0–1 meal/week	≥1 meal/week
Olive oil	Not primary oil	–	Primary oil
Wine	>50 mL/day or ≤1.7 mL/day	1.7–43 mL/day	43-50 mL/day
Red meat and products	≥7 meals/week	4–7 meals/week	<4 meals/week
Fried foods	≥4 meals/week	1–4 meals/week	<1 meal/week
Stick margarine and butter	>30 g/day	15–30 g/day	<15 g/day
Cheese	≥280 g/week	40–280 g/week	<40 g/week
Pastries and desserts	≥140 g/week	100–140 g/week	<100 g/week

− = not applicable.

### PA assessment

Physical activity (PA) was assessed using a self-administered structured questionnaire based on a 7-day recall period. The questionnaire collected detailed information on the intensity, frequency, and duration of PA participation. To standardize reporting, illustrative examples of various PA types were provided to participants. For instance, moderate-intensity physical activity (MPA) was exemplified by activities such as light weightlifting, cycling at a moderate pace, and recreational racquet sports (e.g., tennis or badminton). The survey captured data on the usual weekly frequency (days/week) and session duration (minutes) for vigorous-intensity physical activity (VPA), MPA, and recreation-related PA. Only PA episodes lasting at least 10 consecutive minutes were included in the calculations. VPA was defined as activities that demanded substantial physical effort, resulting in noticeable shortness of breath, significant increases in heart rate, and pronounced sweating, with heavy weightlifting, digging, aerobics, and high-speed cycling as typical examples. MPA was defined as activities that induced mild shortness of breath, moderate increases in heart rate, and light sweating. Total weekly PA time was calculated as the sum of the time spent across all activity types and presented in hours per week.

### Covariates

Residence was classified as urban or rural according to standards issued by the National Bureau of Statistics of China. Educational level was categorized into illiterate, primary, junior high school, and above. Economic status was divided into low, medium, and high groups based on quartiles of annual household income. Marital status was grouped as married or other status (never married, widowed, divorced, separated, or cohabiting). Living alone was defined as a binary variable (yes/no). Current smoking was defined as smoking daily or occasionally at the time of the survey. Current drinking was defined as any alcohol consumption in the past 12 months. Body mass index (BMI) was calculated as weight in kilograms divided by height in meters squared (kg/m^2^). Hypertension was defined as having a systolic blood pressure of ≥140 mmHg, a diastolic blood pressure of ≥90 mmHg, or a self-reported physician diagnosis (22). Diabetes mellitus (DM) was defined as fasting plasma glucose levels of ≥7.0 mmol/L, 2-h postprandial glucose levels of ≥11.1 mmol/L, or a self-reported physician diagnosis (23). Dyslipidemia was defined as having any of the following lipid levels: total cholesterol (TC) of ≥6.22 mmol/L, triglycerides (TG) of ≥2.26 mmol/L, low-density lipoprotein cholesterol (LDL-C) of ≥4.14 mmol/L, or high-density lipoprotein cholesterol (HDL-C) of <1.04 mmol/L (24). Activities of daily living (ADL) disability was defined as difficulty performing any one of the following tasks: dressing, eating, maintaining bowel and bladder control, toileting, bathing, or bed-to-chair transfer (25). Cardio-cerebral disease was defined based on self-reported physician diagnosis of coronary heart disease or stroke and was coded as a binary variable (yes/no). Depression was identified using the 15-item Geriatric Depression Scale (GDS-15), with a cutoff score of ≥ 5 to indicate presence of depressive symptoms (26).

### Statistical analysis

All statistical analyses were performed using SAS version 9.4 (SAS Institute, Cary, NC, United States) and R version 4.5.3 (R Foundation for Statistical Computing, Vienna, Austria). Three types of sampling weights were calculated: a multistage sampling weight, a non-response weight, and a post-stratification weight based on the 2020 Henan population census data for adults aged ≥ 60 years. The final combined weight was calculated by multiplying these three weights together.

Continuous variables were presented as means ± standard deviations (SD) for normally distributed variables or medians (interquartile ranges [IQR]) for skewed variables. Categorical variables were described as *n* (%). Descriptive statistics were based on observed data without imputation. Missing data in covariates were handled using multiple imputation by chained equations (MICE) with 5 imputed datasets and 10 iterations. Rubin’s rules were applied to pool estimates across the imputed datasets. Weighted GLMs were applied to examine the associations between the MIND diet score, PA, and their interactions and MMSE scores across three sequentially adjusted models, where Model 1 was unadjusted. Model 2 was adjusted for age, sex, residence, education level, economic status, marital status, and living alone based on Model 1, and Model 3 was additionally adjusted for current smoking, current drinking, BMI, hypertension, DM, dyslipidemia, cardio-cerebral disease, ADL disability, and depression based on Model 2. We also explored the associations and interactive effects across five individual MMSE domains: orientation, registration, attention and calculation, recall, and language and visual construction skills. Multicollinearity was assessed using the variance inflation factor (VIF) for Models 2 and 3, and no evidence of multicollinearity was detected in either model (Supplementary Table S1). Both the MIND diet score and PA were treated as continuous variables in all models. Sensitivity analyses were performed by retaining all PA data without the extreme-value exclusion, and the interaction between the MIND diet score and PA was re-examined in Model 3. All statistical tests were two-sided, and a *p*-value of < 0.05 was considered statistically significant.

## Results

### Baseline characteristics of the study population

The baseline characteristics of the participants are presented in Table 2. A total of 8,681 participants were included in the final analysis. The majority of the participants were aged 60–69 years (53.5%), were female (53.2%), and resided in rural areas (85.0%). Nearly half of the participants were illiterate (48.1%), while 55.1% had a middle economic status, and 80.4% were married. Current smoking and current drinking were reported by 19.5 and 25.6% of the participants, respectively. Hypertension was the most prevalent chronic condition (76.9%), followed by dyslipidemia (46.8%), DM (37.7%), cardio-cerebral disease (29.6%), and ADL disability (18.3%). The mean BMI was 25.4 ± 3.6 kg/m^2^, the mean MMSE score was 23.32 ± 5.50, the mean MIND diet score was 3.90 ± 1.58, and the median weekly PA was 6.0 h (IQR: 1.5, 14.0).

**Table 2:** Baseline characteristics of the study participants.

Characteristics	Sample, *n* (%)^a^
Total	8,681(100.0)
Age group, *n* (%)	
60–69	4,574 (53.5)
70–79	3,060 (32.6)
≥80	1,047 (13.8)
Sex, *n* (%)	
Male	4,082 (46.8)
Female	4,599 (53.2)
Residence, *n* (%)	
Urban	1,977 (15.0)
Rural	6,704 (85.0)
Education level, *n* (%)	
Illiterate	3,920 (48.1)
Primary school	1,298 (14.9)
Junior high school	2,176 (23.3)
High school or above	1,287 (13.7)
Economic status*, *n* (%)	
Low	1,504 (27.2)
Middle	3,247 (55.1)
High	1,307 (17.7)
Marital status, *n* (%)	
Married	6,935 (80.4)
Unmarried/others	1,746 (19.6)
Living alone, *n* (%)	
Yes	1,454 (17.3)
No	7,227 (82.7)
Current smoking, *n* (%)	
Yes	1,696 (19.5)
No	6,985 (80.5)
Current drinking, *n* (%)	
Yes	2,177 (25.6)
No	6,504 (74.4)
Depression, *n* (%)	
Yes	4,351 (50.3)
No	4,330 (49.7)
Hypertension*, *n* (%)	
Yes	6,653 (76.9)
No	2,022 (23.1)
DM*, *n* (%)	
Yes	3,177 (37.7)
No	4,870 (62.3)
Dyslipidemia*, *n* (%)	
Yes	4,095 (46.8)
No	4,570 (53.2)
Cardio-cerebral disease, *n* (%)	
Yes	2,729 (29.6)
No	5,952 (70.4)
ADL disability, *n* (%)	
Yes	1,618 (18.3)
No	7,063 (81.7)
BMI (kg/m^2^), mean ± SD	25.4 ± 3.6
MMSE score, mean ± SD	23.32 ± 5.50
MIND diet score, mean ± SD	3.90 ± 1.58
PA (h/week), median (IQR)	6.0 (1.5, 14.0)

a Percentages are weighted.

Values are unweighted counts (*n*). *Missing data were present for economic status (*n* = 2,623), diabetes mellitus (*n* = 634), dyslipidemia (*n* = 16), and hypertension (*n* = 6). The descriptive statistics presented here are based on observed data without imputation. DM, diabetes mellitus; BMI, body mass index; ADL, activities of daily living; PA, physical activity; IQR, interquartile range; MMSE, Mini-Mental State Examination; SD, standard deviation.

### Associations between the MIND diet score, PA, and their interactions and cognitive function

The associations between the MIND diet score, PA, and their interactions with MMSE scores are shown in Table 3. In the unadjusted Model 1, both the MIND diet score (*β* = 0.756, 95% *CI*: 0.626–0.886, *p* < 0.001) and PA (*β* = 0.072, 95% *CI*: 0.031–0.114, *p* < 0.001) were positively associated with MMSE scores. A significant negative interaction between the MIND diet score and PA was observed (*β* = −0.011, 95% *CI*: −0.020 to −0.002, *p* = 0.015). After adjusting for demographic variables in Model 2, the main effect of the MIND diet score remained statistically significant (*β* = 0.321, 95% *CI*: 0.213–0.430, *p* < 0.001), while the main effect of PA became non-significant (*β* = 0.025, 95% *CI*: −0.009 to 0.059, *p* = 0.147). The interaction term was attenuated and was no longer statistically significant (*β* = −0.006, 95% *CI*: −0.014 to 0.002, *p* = 0.116). In the fully adjusted Model 3, the MIND diet score was associated with higher cognitive function (*β* = 0.259, 95% *CI*: 0.154–0.364, *p* < 0.001), while PA still showed no significance (*β* = 0.016, 95% *CI*: −0.017 to 0.049, *p* = 0.335), and no significant interaction was observed (*β* = −0.005, 95% *CI*: −0.013 to 0.002, *p* = 0.152).

**Table 3:** Interaction between the MIND diet score and PA on MMSE scores.

Variables	Model 1			Model 2			Model 3		
	Weighted *β* (95% *CI*)	*SE*	*p*-value	Weighted *β* (95% *CI*)	*SE*	*P*-value	Weighted *β* (95% *CI*)	*SE*	*p*-value
MIND diet score	0.756 (0.626, 0.886)	0.066	<0.001	0.321 (0.213, 0.430)	0.055	<0.001	0.259 (0.154, 0.364)	0.054	<0.001
PA	0.072 (0.031, 0.114)	0.021	<0.001	0.025 (−0.009, 0.059)	0.017	0.147	0.016 (−0.017, 0.049)	0.017	0.335
MIND diet ×PA	−0.011(−0.020,-0.002)	0.005	0.015	−0.006 (−0.014, 0.002)	0.004	0.116	−0.005 (−0.013, 0.002)	0.004	0.152

Model 1: Unadjusted. Model 2: Adjusted for demographic variables including sex, age, residence, educational level, economic status, marital status, and living alone. Model 3: Additionally adjusted for current smoking, current drinking, BMI, hypertension, DM, dyslipidemia, cardio-cerebral disease, ADL, and depression based on Model 2.

### Associations between the MIND diet score and PA and five cognitive domains

We further examined the associations between the MIND diet score and PA with five individual cognitive domains using fully adjusted weighted linear regression models (Table 4). The MIND diet score showed significant positive associations with registration (*β* = 0.019, 95% *CI*: 0.003–0.036, *p* = 0.022), attention and calculation (*β* = 0.050, 95% *CI*: 0.011–0.088, *p* = 0.011), and recall (*β* = 0.043, 95% *CI*: 0.020–0.066, *p* < 0.001). For orientation as well as language and visual construction skills, no significant associations were observed for the MIND diet score (all *p* > 0.05). PA showed no significant association with any of the cognitive domains (all *p* > 0.05). Additionally, the interactions between the MIND diet score and PA were also non-significant across all five domains (all *p* > 0.05).

**Table 4:** Interaction between the MIND diet score and PA on five separate domains.

Domains	Variables	Weighted *β* (95% *CI*)	*SE*	*p*-value
Orientation	MIND diet score	0.034 (−0.100, 0.168)	0.068	0.620
	PA	0.009 (−0.036, 0.053)	0.023	0.706
	MIND diet × PA	−0.004 (−0.013, 0.006)	0.005	0.440
Registration	MIND diet score	0.019 (0.003, 0.036)	0.008	0.022
	PA	0.002 (−0.003, 0.007)	0.003	0.435
	MIND diet × PA	−0.001 (−0.002, 0.000)	0.001	0.261
Attention and calculation	MIND diet score	0.050 (0.011, 0.088)	0.020	0.011
	PA	0.003 (−0.009, 0.015)	0.006	0.616
	MIND diet × PA	−0.001 (−0.004, 0.001)	0.001	0.295
Recall	MIND diet score	0.043 (0.020, 0.066)	0.012	<0.001
	PA	0.004 (−0.003, 0.011)	0.004	0.283
	MIND diet × PA	−0.001 (−0.003, 0.001)	0.001	0.260
Language and visual construction skills	MIND diet score	0.050 (−0.058, 0.159)	0.055	0.364
	PA	0.003 (−0.036, 0.041)	0.020	0.898
	MIND diet × PA	−0.003 (−0.011, 0.005)	0.004	0.498

*All estimates were obtained from the weighted linear regression model (Model 3) with multiple imputation (m = 5) and Rubin’s rules for pooling. Model 3 was adjusted for age, sex, residence, education level, economic status, marital status, living alone, current smoking, current drinking, BMI, hypertension, DM, dyslipidemia, cardio-cerebral disease, ADL, and depression. SE, standard error. Estimates with absolute values < 0.0005 are reported as 0.000 due to rounding. PA, physical activity; BMI, body mass index; DM, diabetes mellitus; ADL, activities of daily living.

### Sensitivity analysis

In the sensitivity analysis retaining all PA data without the extreme-value exclusion, the MIND diet score remained significantly associated with cognitive function (*β* = 0.248, 95% *CI*: 0.156–0.339, *p* < 0.001), whereas PA showed no significant association (*β* = 0.012, 95% *CI*: −0.007–0.030, *p* = 0.208). The interaction between the MIND diet score and PA was borderline statistically significant (*β* = −0.004, 95% *CI*: −0.008–0.000, *p* = 0.049) (see Table 5).

**Table 5:** Sensitivity analysis with all PA data retained (no extreme-value exclusion).

Variables	Weighted *β* (95% *CI*)	*SE*	*p*-value
MIND diet score	0.248 (0.156, 0.339)	0.047	<0.001
PA	0.012 (−0.007, 0.030)	0.009	0.208
MIND diet score × PA	−0.004 (−0.008, 0.000)	0.002	0.049

Model 3: Adjusted for age, sex, residence, education level, economic status, marital status, living alone, current smoking, current drinking, BMI, hypertension, DM, dyslipidemia, cardio-cerebral disease, ADL, and depression.

## Discussion

In this cross-sectional study of community-dwelling older adults in central China, we found that a higher MIND diet score was independently associated with better cognitive function. However, PA showed no significant independent association with cognitive outcomes. In the main analysis, no significant interaction was observed between the MIND diet score and PA, whereas in the sensitivity analysis, the interaction was borderline statistically significant. This inconsistency suggests that the evidence for an interaction effect remains inconclusive and controversial. Future studies using objective PA measures are needed to clarify whether PA truly modifies the association between the MIND diet score and cognitive function.

Our finding that the MIND diet score is associated with better cognitive function is consistent with a growing body of evidence from both Western and Asian populations (27–30). Notably, a recent systematic review by Tse et al. (31) reported that most observational studies support our findings, whereas randomized evidence remains mixed. Similarly, a 3-year randomized controlled trial found no significant differences in cognition or brain magnetic resonance imaging (MRI) outcomes between the MIND diet and control groups (32). These findings suggest that while observational evidence supports a positive association between MIND diet adherence and cognitive function, further interventional studies are required to clarify whether this association reflects a causal effect.

The neuroprotective effects of the MIND diet are supported by multiple biological pathways. The diet is rich in antioxidants, including vitamin E, vitamin C, carotenoids, and polyphenols, which neutralize reactive oxygen species and reduce oxidative stress, a key contributor to amyloid-β plaque formation and tau phosphorylation (33, 34). In parallel, its anti-inflammatory components, particularly omega-3 fatty acids, downregulate microglial activation and pro-inflammatory signaling pathways such as nuclear factor kappa B, which has been associated with reduced amyloid-β production. Folate, abundant in green leafy vegetables, may further contribute by lowering plasma homocysteine levels, a risk factor for cognitive decline (33). Adherence to the MIND diet has also been associated with improved cerebrovascular function, including reduced white matter hyperintensities and a lower likelihood of small vessel arteriosclerosis (35). Emerging evidence further suggests that the MIND diet may promote cognitive resilience through modulation of the gut microbiota via the microbiota–gut–brain axis (36). Although the exact mechanisms remain to be fully elucidated, these multi-pathway actions provide a biologically plausible foundation for the observed association between adherence to the MIND diet and better cognitive function in older adults.

Contrary to our initial hypothesis and the findings of Chen et al. (37), which reported a protective effect of PA on cognition, we found no significant association between PA and cognitive function. The apparent protective effect observed in the unadjusted model was completely attenuated by sociodemographic characteristics, suggesting that it might be largely explained by confounding factors such as age, education, and socioeconomic status. This interpretation is supported by a study among multi-ethnic older adults in Malaysia, which reported that the association between PA and cognitive function was fully attenuated after adjusting for education and socioeconomic status (38). Together, these findings suggest that the previously reported protective effect of PA on cognition may be substantially confounded by sociodemographic factors. Additionally, reliance on self-reported PA measures may have attenuated the true association due to recall bias. Future investigations incorporating objective PA monitoring, such as wrist-worn accelerometers or pedometers, could provide more precise estimates of activity volume and intensity and further clarify whether PA is independently associated with cognitive health in this population.

We further investigated the interaction between the MIND diet score and PA in relation to MMSE scores, but no significant association was observed. This finding is consistent with the findings from the Rotterdam study, a large-scale prospective cohort study. This study found that both higher PA and greater adherence to the MIND diet were independently associated with a reduced risk of dementia, but no significant interaction was found on either the additive scale or the multiplicative scale (39). In contrast to our findings, two previous studies reported significant interactions between the MIND diet score and PA in relation to cognitive outcomes. One study of 132 healthy older adults found a significant interaction for executive functioning and gray matter volume, with low levels of both being associated with disproportionately poorer outcomes (40). Another study based on the Rush Memory and Aging Project found that individuals with the lowest MIND diet adherence experienced greater cognitive benefits from PA than those with higher adherence (41). These discrepancies may be attributable to differences in PA assessments. Notably, the latter study used objective actigraphy, whereas our study and the former relied on self-reported measures. Objective measurement may be more sensitive to detecting subtle interaction effects, whereas self-reported measures may introduce measurement error that could bias the estimates. Additionally, differences in outcome measures, sample characteristics, and cognitive assessment tools may have contributed to the inconsistent findings. Taken together, these findings highlight the need for standardized methodologies and objective PA assessments in future studies to clarify the nature of the diet–PA interaction in cognitive aging. We also explored whether the associations varied across five cognitive domains. The MIND diet score showed significant positive associations with registration, attention and calculation, and recall but not with orientation or language and visual construction skills. Neither PA nor the MIND diet score-PA interaction term was significantly associated with performance in any cognitive domain. These domain-specific findings were consistent with the main analysis and suggest that the MIND diet score may be associated with certain cognitive functions, particularly memory and attention. Further studies are warranted to confirm these findings.

This study has several strengths. To the best of our knowledge, this is the first study to simultaneously examine the independent and interactive effects of the MIND diet and PA on both global and domain-specific cognitive function in a large sample of older adults in central China. In addition, we adjusted for a wide range of sociodemographic characteristics, lifestyle factors, and multiple chronic diseases, ensuring the robustness of our results. Several limitations should also be acknowledged. First, the cross-sectional design cannot establish causal relationships between the MIND diet, PA, and cognitive function. Second, dietary intake and PA were self-reported, which may be subject to measurement error and recall bias. Future studies using objective measures such as accelerometers for PA and nutritional biomarkers for dietary intake are warranted. Third, we did not have data on medication use or family history of dementia, which may be potential confounders. Fourth, all participants were aged ≥60 years, and the findings may not be generalized to middle-aged and younger populations.

From a public health perspective, our findings provide practical guidance for cognitive health management among older adults in central China. The MIND diet offers a practical, affordable, and feasible approach to preserving cognitive function, especially in populations with limited resources or those with insufficient PA. Notably, however, our results do not deny the established benefits of PA, which is known to improve cardiovascular fitness and maintain physical function in older adults.

## Conclusion

Higher adherence to the MIND diet was independently associated with better cognitive function among community-dwelling older adults in Central China. These findings suggest that adherence to the MIND diet may be a practical strategy for promoting cognitive health among older adults.

## Data Availability

The raw data supporting the conclusions of this article will be made available by the authors, without undue reservation.
