# Supplementary material for: Mediterranean-DASH intervention for neurodegenerative delay diet adherence, physical activity, and cognitive function among community-dwelling older adults
Source: Front Nutr. 2026 Oct 28;13:1947659. doi: 10.3389/fnut.2026.1947659 (PMC13752176; doi:10.3389/fnut.2026.1947659)
Supplement: Supplementary file 1 [file Table_1.docx]

**Supplementary Table S1.** Multicollinearity diagnostics (VIF) for Model 2 and Model 3.

|  | Model 2 | | | Model 3 | | |
| --- | --- | --- | --- | --- | --- | --- |
| Variable | GVIF | Df | GVIF^(1/(2×Df)) | GVIF | Df | GVIF^(1/(2×Df)) |
| MIND Diet score | 1.93 | 1 | 1.39 | 1.95 | 1 | 1.40 |
| PA | 7.05 | 1 | 2.66 | 7.08 | 1 | 2.66 |
| MIND Diet × PA | 8.33 | 1 | 2.89 | 8.34 | 1 | 2.89 |
| Sex | 1.15 | 1 | 1.07 | 1.17 | 1 | 1.08 |
| Age group | 1.14 | 2 | 1.03 | 1.18 | 2 | 1.04 |
| Residence | 1.25 | 1 | 1.12 | 1.27 | 1 | 1.13 |
| Education level | 1.31 | 2 | 1.07 | 1.32 | 2 | 1.07 |
| Economic status | 1.41 | 2 | 1.09 | 1.44 | 2 | 1.10 |
| Marital status | 1.35 | 1 | 1.16 | 1.36 | 1 | 1.16 |
| Living alone | 1.32 | 1 | 1.15 | 1.32 | 1 | 1.15 |
| BMI | — | — | — | 1.09 | 1 | 1.04 |
| Hypertension | — | — | — | 1.07 | 1 | 1.03 |
| DM | — | — | — | 1.07 | 1 | 1.04 |
| Dyslipidemia | — | — | — | 1.10 | 1 | 1.05 |
| Cardio-cerebral disease | — | — | — | 1.06 | 1 | 1.03 |
| ADL | — | — | — | 1.10 | 1 | 1.05 |
| Depression | — | — | — | 1.09 | 1 | 1.04 |

Note: Adjusted VIF = GVIF^(1/(2×Df)). All adjusted VIFs for covariates were below 1.5, below the conventional threshold of 5, indicating no substantial multicollinearity. The elevated values for PA and its interaction term are expected due to the mathematical correlation between product terms and their constituent main effects. PA, physical activity; BMI: Body mass index; DM,Diabetes mellitus; ADL:Activities of daily living.
